# Triacylglycerol composition, physico-chemical characteristics and oxidative stability of interesterified canola oil and fully hydrogenated cottonseed oil blends

**DOI:** 10.1186/s12944-015-0140-0

**Published:** 2015-10-29

**Authors:** Muhammad Imran, Muhammad Nadeem

**Affiliations:** Institute of Home and Food Sciences, Faculty of Science and Technology, Government College University, Faisalabad, Pakistan; Department of Dairy Technology, University of Veterinary and Animal Sciences, Lahore, Pakistan

**Keywords:** Canola oil, Hydrogenated cottonseed oil, Interesterification, Fat-based products, TAG, Storage stability, Discerning consumers

## Abstract

**Background:**

Partial hydrogenation process is used worldwide to produce shortening, baking, and pastry margarines for food applications. However, demand for such products is decreased during last decade due to their possible links to consumer health and disease. This has raised the need to replace hydrogenation with alternative acceptable interesterification process which has advantage in context of modifying the physico-chemical properties of edible fat-based products. Therefore, the main mandate of research was the development of functional fat through chemical interesterification of canola oil (CaO) and fully hydrogenated cottonseed oil (FHCSO) mixtures.

**Methods:**

Blends were prepared in the proportions of 75:25 (T_1_), 50:50 (T_2_) and 25:75 (T_3_) of CaO:FHCSO (w/w). Interesterification was performed using sodium methoxide (0.2 %) as catalyst at 120 °C, under reduced pressure and constant agitation for 60 minutes. The non-interesterified and interesterified CaO:FHCSO blends were evaluated for triacylglycerol (TAG) composition, physico-chemical characteristics, oxidative stability and consumer acceptability at 0, 30 and 60 days of storage interval.

**Results:**

The oleic acid (58.3 ± 0.6 %) was predominantly present in CaO while the contents of stearic acid (72 ± 0.8 %) were significantly higher in FHCSO. Maximum trisaturated (S_3_) contents (63.9 ± 0.5 %) were found in T_3_ while monounsaturated (S_2_U), diunsaturated (U_2_S) and triunsaturated (U_3_) contents were quite low in T_2_ and T_3_ before interesterification. A marked reduction in S_3_ and U_3_ contents with concomitant increase in S_2_U and U_2_S contents was observed for all CaO:FHCSO blends on interesterification. During storage, the changes in S_3_, S_2_U and U_2_S contents were not found significant *(p ≥ 0.05)*. However, maximum decrease 13 %, 7.5 and 5.6 % in U_3_ contents for T_1_, T_2_ and T_3_ was noted after 60-days of interesterification, respectively. The Lovibond color R, melting point, refractive index, specific gravity, peroxide and free fatty acids values of CaO:FHCSO blends decreased after interesterification and increased within the permissible limits during storage *(p ≥ 0.05)*. The CaO:FHCSO blends maintained their sensory acceptability before and after interesterification which decreased significantly as storage length increased from days 30 to 60-days. Most important was the 50 % CaO:50 % FHCSO blend (T_2_) which possessed the desirable TAG profile, physico-chemical and sensory characteristics coming from T_1_ and T_3_.

**Conclusions:**

The present study concludes that functional lipids with desirable characteristics can be developed through interesterification of 50 % CaO:50 % FHCSO blend and should be explored as ingredient for the production of various healthier products for discerning consumers.

## Background

Lipids constitute an important component of human diet and have many important functions in the human health and disease. Generally, they are found in every cell membrane of biological system and serve as a source of essential fatty acids such as linoleic acid and α-linolenic acid, vehicles for fat-soluble vitamins (A, D, E and K), and provision of energy. The consumption of omega-3 fatty acids in foods tends to block the development of atherosclerosis, reduces the number of subsequent heart attacks, interferes with abnormal clotting of the blood, and coronary thrombosis. They may be active also against certain forms of cancer, immunological diseases, hypertension and hyperlipidemic conditions [1]. Of particular importance to nutrition and culinary specialists is that lipids provide satiety through their aroma, taste and texture. When fats and oils are eliminated in cooking and baking, the final products can be tasteless and unsatisfying [2, 3]. The scientific community agrees that fats and oils enhance the texture and palatability of foods through their wide range of melting and crystallization properties. Interesterification, hydrogenation and fractionation are three processes available to food manufactures to tailor the physical, functional and chemical properties of food lipids. Approximately, 10 % of all edible oils and fats in the world are fractionated or interesterified while one-third is fully hydrogenated [4, 5].

Partial hydrogenation is a chemical process leading to the saturation of double bonds to harden fats for use as margarines and shortening base stocks. Unfortunately, it has been determined that partial hydrogenation of vegetable oils is the main source of *trans* fatty acids in fried or processed foods. High intake of *trans* fatty acids as compared to saturated fatty acids increase the level of LDL-cholesterol, total cholesterol and triglycerides in the bloodstream which lead toward high risk of atherosclerosis and coronary heart disease [6, 7]. This has raised the need to replace partially hydrogenated fats with alternative acceptable fats. Consequently, industries involved in food processing are looking for alternative to partial hydrogenation for making shortening and hard fats [8, 9]. Chemical interesterification is of major importance to edible oil industry for the preparation of plastic fat with low *trans* isomer contents or even with the absence of these compounds and providing the functional properties necessary to meet the challenges of today’s food industry [10]. Chemical interesterification is a relatively straightforward reaction which consist of catalyst activation, ester bond cleavage and fatty acid interchange steps. Interesterification process causes fatty acids redistribution within and among triacylglycerol molecules which can lead to substantial changes in lipid functionality. Commercially, the interesterification process is regularly used in processing fully hydrogenated vegetable oil and liquid oil blends to produce a variety of margarines, frying oils and confectionery fats [11]. Important factors that determine the application of such interesterified fats include fatty acids composition, physico-chemical characteristics, oxidative stability and consumer acceptability [12]. The situation demands to explore the potential of chemical interesterification process for development of modified functional fat with desirable composition for discerning consumers using commercially available vegetable oils. CaO stands as interesting substrate with comprehensive unsaturated fatty acids composition and free of *trans* fatty acids while FHCSO shows desirable characteristics for the production of interesterified fat base with considerable saturated fatty acids concentration. The main objectives of study were to change the overall physical, chemical and sensory profile of CaO and FHCSO blends by chemical interesterification process.

## Methods

### Raw materials

The seeds of canola were procured from Oilseeds Research Institute, Faisalabad, Pakistan. The unprocessed seeds were cleaned to remove any debris or field dirt and any other extraneous matters. CaO, obtained by pressing the seeds in mini oil presser (china made; capacity 2–3 kg/h), was bleached by heating it to 110 °C, mixing with clay (bentonite) for 30 min and filtered. Refined and deodorized CaO was cooled and stored in Tin cans of 1-kg capacity (0.20 mm sheet thickness with flange of 2-mm) at 5 ± 1 °C. Refined, bleached and deodorized FHCSO was obtained from United Industries, Ltd. Kashmir Road, Faisalabad while sodium methoxide (Merck) was purchased from a scientific supplier of Lahore, Pakistan.

### Blend preparation

FHCSO was supplemented into Cao at three different levels i.e. T_1_ (75 % CaO and 25 % FHCSO), T_2_ (50 % CaO and 50 % FHCSO) and T_3_ (25 % CaO and 75 % FHCSO). Each of the prepared blends was placed into a round bottom flask (1000 mL). For an optimum interesterification reaction, the oil portion (500 mL) was neutralized and oven dried at 60 °C to ensure a good result for catalyst consumption.

### Chemical interesterification process

Chemical interesterification of treatments was carried out according to the method of Grimaldi et al. [13] with some modifications. Sodium methoxide was used as catalyst (0.2 %) for chemical interesterification. When reaction temperature of sample reached the 120 °C, sodium methoxide was added under reduced pressure and constant agitation for 60 min. The temperature was then decreased to 90 °C. To end the reaction with distinctive dark brown color, an excess of citric acid (20 %) was added to neutralize the catalyst. The excess of citric acid and sodium methoxide was removed with warm water washes and the samples were vacuum-dried. Residual water was removed with an excess of anhydrous sodium sulfate (15 %), followed by filtration through Whatman # 2 filter paper and oven dried at 60 °C.

### Triacylglycerol composition

The triacylglycerol composition of experimental samples was determined by the method Ce 1f–96 given in AOCS [14]. The oil sample (50 μL) was methyated in the presence of 4 mL KOH (1 M) at room temperature for 1 h in order to convert fatty acids into their respective methyl esters. The resultant fatty acid methyl esters (FAMEs) were extracted with GC grade n–hexane and analyzed by Gas Chromatograph (Varian 3900) apparatus equipped with an auto sampler, flame–ionization detector (FID) and supelco wax column (30 m × 0.25 μm film coating). The samples (1 μL) were injected with Helium (1 mL/min) as a carrier gas onto the column, which was programmed for operating conditions such as column oven temperature 160 °C @ 0 min with subsequent increase of 3 °C/min until 180 °C. The column oven temperature was increased from 180 °C to 220 °C @ 1 °C/min and was held for 7.5 min at 220 °C. Split ratio was 50 % with injector 240 °C and detector 250 °C temperatures. The peak areas and triglycerides composition were calculated for each sample by retention time using Varian Chem Station software. The standards of triglyceride methyl esters purchased from Sigma-Aldrich were also run under the same conditions for comparison with experimental samples.

### Physico-chemical and oxidative parameters

The color (R value) of non-interesterified and interesterified samples was determined by Lovibond Tintometer using 5.25" quartzcell. Melting point of samples was assessed by AOAC [15] Method No. 920–157. The refractive index of blends was recorded by means of Abbe’s refractometer at room temperature following the protocol No. Cc 7–25 as described in AOCS [14]. Specific gravity of samples was measured at 25 °C with specific gravity bottle (Pycnometer, Sigma-Aldrich) according to AOCS [14] Method No. Cc 10a–25. Saponification value (Method No. Cd 3–25), iodine value (Method No. Cd 1d–92), peroxide value (Method No. Cd 8–53) and free fatty acid value (Method No. Ca 5a–40) of CaO:FHCSO blends were estimated by following AOCS [14], respectively.

### Sensory evaluation

Fourteen panel judges consisting of experienced and untrained panelists carried out the sensory analysis of samples according to the instructions given by Meilgaard et al. [16]. Each judge gave written informed consent after explanation of risks and benefits of participation prior to the study. The panelists were provided informative instructions and brief definitions of attributes such as appearances, flavor and overall acceptability. Each panelist received the samples assigned with random three–digit code numbers. Each panelist was asked to list their preference on a 9–cm comparison line (1 = dislike extremely to 9 = like extremely). The sensory analysis was performed and completed at 0, 30 and 60 days of storage interval for experimental treatments.

### Statistical analysis

The data obtained for each parameter was subjected to statistical analysis to determine the level of significance by using the software package (Statistic 8.1) according to the method described [17]. The Duncan’s multiple range (DMR) test was used to estimate the level of significance that existed between the mean values.

## Results and discussion

### Fatty acids composition and physico-chemical characteristics of raw materials

The raw materials, CaO and FHCSO, were analyzed for their fatty acids composition and physico-chemical parameters before CaO:FHCSO blend preparation. The oil contents in canola seed were found 40.5 ± 2.8 %. The oleic (58.3 ± 0.6 %), linoleic (22.8 ± 0.5 %), α-linolenic (9.7 ± 0.4 %), palmitic (4.5 ± 0.3 %) and stearic acids (1.6 ± 0.2 %) were predominantly present in CaO. Refined and bleached CaO showed Lovibond color R value (1.48 ± 0.14), melting point (−9 ± 1 °C), refractive index (1.465 ± 0.002), specific gravity (0.921 ± 0.001), saponification value (188 ± 3), iodine value (122 ± 3), peroxide value (0.165 ± 0.008 meq/kg) and free fatty acids value (0.1 ± 0.004 %). The contents of stearic acid (72 ± 0.8 %) were found significantly higher in FHCSO obtained from local Vanaspati manufacturing industry followed by palmitic acid (20 ± 0.5 %) and oleic acid (4 ± 0.3 %). FHCSO possessed Lovibond color R value (2.1 ± 0.22), melting point (59 ± 1 °C), refractive index (1.472 ± 0.003), specific gravity (0.918 ± 0.001), saponification value (198 ± 2), iodine value (6 ± 2), peroxide value (0.167 ± 0.006 meq/kg) and free fatty acids value (0.098 ± 0.003 %).

### TAG composition of CaO:FHCSO blends

The TAG profile is known as potential key for the understanding of several physico-chemical properties of given oil or fat developed through modification process. The major TAG classification (trisaturated = S_3_; monounsaturated = S_2_U; diunsaturated = U_2_S; triunsaturated = U_3_) of Cao:FHCSO blends before and after interesterification process at different storage days has been presented in Table 1. The results show that addition of FHCSO to CaO before interesterification increased the saturated fatty acids contents in different blends. Maximum S_3_ contents (63.9 ± 0.5 %) were found in T_3_ while S_2_U, U_2_S and U_3_ contents were quite low in T_2_ and T_3_ when were directly compared with T_1_ before interesterification of Cao:FHCSO blends. A marked reduction in S_3_ and U_3_ contents was observed for all experimental treatments on completion of interesterification. On the other hand, S_2_U and U_2_S contents of T_1_, T_2_ and T_3_ increased significantly after interesterification. Maximum increment in U_2_S contents was recorded for T_1_ with highest value (50.4 ± 0.5 %)_._ There are many reports on effects of interesterification on TAG composition of the end product which evident that the concentrations of several TAG were increased, some were decreased, and several new TAG was formed [18]. The randomization process causes rearrangement of TAG species, reduction of S_3_ and U_3_ contents and increase in S_2_U and U_2_S TAGs [19–25]. After interesterification, the high proportions of S_3_ present in the starting blends were reduced 73–89 % and greatest changes were observed for the blends with 40–50 % hard stock (a relative decrease of U_3_ of 38–64 % and a relative increase of U_2_S of 59–130 % for different edible oil blends [26]. Total unsaturated (U)/Total saturated (S) ratio increased significantly from their initial value after interesterification with order T_1_ (28.3 ± 0.3 %) > T_2_ (3.06 ± 0.2 %) > T_3_ (1.43 ± 0.1 %). The U/S ratios for interesterified Cao:FHCSO blends were higher than 1 and conformed to the recommendation of the Food and Agricultural Organization/World Health Organzation (FAO/WHO) and European Union Committee (EUC) for a minimal unsaturated fatty acids/saturated fatty acids ratio [26]. During storage, the changes in S_3_, S_2_U and U_2_S contents were not found significant *(p ≥ 0.05)*. However, maximum decrease 13, 7.5 and 5.6 % in U_3_ contents for T_1_, T_2_ and T_3_ was noted after 60-days of interesterification, respectively. Similarly, the unsaturated fatty acids/saturated fatty acids ratios of refined cottonseed oil and virgin olive oil blend samples after chemical interesterification showed slight reduction during 28 days storage at 60 °C [27]. The zero-*trans* margarine manufactured from various liquid oils blends using 0.5 % sodium methoxide catalyst at 70 °C and vigorous agitation for 15 min appeared to remain preserved after interesterification [28]. The total TAG contents of all experimental treatments slightly decreased after interesterification and significantly during storage *(p ≤ 0.05)* which may indicate the production of partial mono- and diacylglycerols in Cao:FHCSO blends. Similar results were observed by Kowalski et al. [29]. The desirable increments in S_2_U and U_2_S contents of T_2_ point toward its better TAG composition as compared to T_1_ and T_3_. Specific structured lipids developed through TAG modification by interesterification have received increasing attention for treatment of nutritional disorders through their absorption, metabolism and distribution pattern into biological tissues and this may provide useful information for the preparation for dietary supplements with specific functions [30, 31].

**Table 1:** Effect of interesterification process on triacylglycerol classes of canola oil and fully hydrogenated cottonseed oil blends

Triacylglycerol (TAG, %)	Experimental treatment	Processing and storage time			
		Before IE	After IE		
			0 day	30 days	60 days
Trisaturated (S_3_)	T_1_	18.6 ± 0.2^a^	3.3 ± 0.1^b^	4.3 ± 0.1^b^	5.1 ± 0.2^b^
	T_2_	42.4 ± 0.4^a^	23.7 ± 0.3^b^	24.3 ± 0.3^b^	25.1 ± 0.4^b^
	T_3_	63.9 ± 0.5^a^	39.6 ± 0.4^b^	40.4 ± 0.4^b^	41 ± 0.3^b^
Monounsaturated (S_2_U)	T_1_	1.1 ± 0.1^b^	10.2 ± 0.2^a^	11.4 ± 0.2^a^	12.5 ± 0.2^a^
	T_2_	0.7 ± 0.1^b^	17.8 ± 0.2^a^	18.3 ± 0.3^a^	19 ± 0.3^a^
	T_3_	0.5 ± 0.1^b^	21.4 ± 0.3^a^	21.8 ± 0.2^a^	22.2 ± 0.3^a^
Diunsaturated (U_2_S)	T_1_	16.8 ± 0.2^b^	50.4 ± 0.5^a^	49.2 ± 0.4^a^	48.3 ± 0.4^a^
	T_2_	9.4 ± 0.2^b^	29.5 ± 0.3^a^	29.1 ± 0.3^a^	28 ± 0.4^a^
	T_3_	5.5 ± 0.1^b^	19.6 ± 0.2^a^	19 ± 0.2^a^	18.3 ± 0.3^a^
Triunsaturated (U_3_)	T_1_	63.1 ± 0.6^a^	32.9 ± 0.3^b^	30.4 ± 0.4^c^	28.6 ± 0.3^d^
	T_2_	46.7 ± 0.4^a^	25.4 ± 0.3^b^	24.2 ± 0.4^c^	23.5 ± 0.3^d^
	T_3_	29.5 ± 0.3^a^	15.9 ± 0.2^b^	15.5 ± 0.2^c^	15 ± 0.2^d^
Total Saturated (S)	T_1_	18.6 ± 0.2^a^	3.3 ± 0.1^b^	4.3 ± 0.1^b^	5.1 ± 0.2^b^
	T_2_	42.4 ± 0.4^a^	23.7 ± 0.3^b^	24.3 ± 0.3^b^	25.1 ± 0.4^b^
	T_3_	63.9 ± 0.5^a^	39.6 ± 0.4^b^	40.4 ± 0.4^b^	41 ± 0.3^b^
Total Unsaturated (U)	T_1_	81 ± 0.6^d^	93.5 ± 0.7^a^	91 ± 0.8^b^	89.4 ± 0.7^c^
	T_2_	56.8 ± 0.6^d^	72.7 ± 0.6^a^	71.6 ± 0.8^b^	70.5 ± 0.7^c^
	T_3_	35.5 ± 0.4^d^	56.9 ± 0.5^a^	56.2 ± 0.5^b^	55 ± 0.6^c^
U/S ratio	T_1_	4.35 ± 0.2^d^	28.3 ± 0.3^a^	21.1 ± 0.3^b^	17.5 ± 0.2^c^
	T_2_	1.33 ± 0.2^d^	3.06 ± 0.2^a^	2.94 ± 0.3^b^	2.80 ± 0.3^c^
	T_3_	0.55 ± 0.1^d^	1.43 ± 0.1^a^	1.36 ± 0.2^b^	1.29 ± 0.2^c^
Total TAG^e^	T_1_	99.6 ± 0.3^a^	96.8 ± 0.6^b^	95.3 ± 0.8^b^	94.5 ± 0.8^c^
	T_2_	99.2 ± 0.4^a^	96.4 ± 0.5^b^	95.9 ± 0.6^b^	95.6 ± 0.7^b^
	T_3_	99.4 ± 0.4^a^	96.5 ± 0.7^b^	95.7 ± 0.8^b^	95.3 ± 0.9^b^

IE: Interesterification; T_1_ (75 % CaO:25 % FHCSO); T_2_ (50 % CaO:50 % FHCSO); T_3_ (25 % CaO:75 % FHCSO); Total saturated (S) calculated by Saturated (S_3_); Total Unsaturated (U) calculated by Monounsaturated (S_2_U) + Diunsaturated (U_2_S) + Triunsaturated (U_3_); Values represent the mean ± standard deviation; *n* = 3

^a, b, c, d^Means in a row with different superscripts were significantly different (*p* < 0.05)

^e^Total TAG calculated by Saturated (S_3_) + Monounsaturated (S_2_U) + Diunsaturated (U_2_S) + Triunsaturated (U_3_)

### Physico-chemical characteristics of CaO:FHCSO blends

**Fig. 1:**
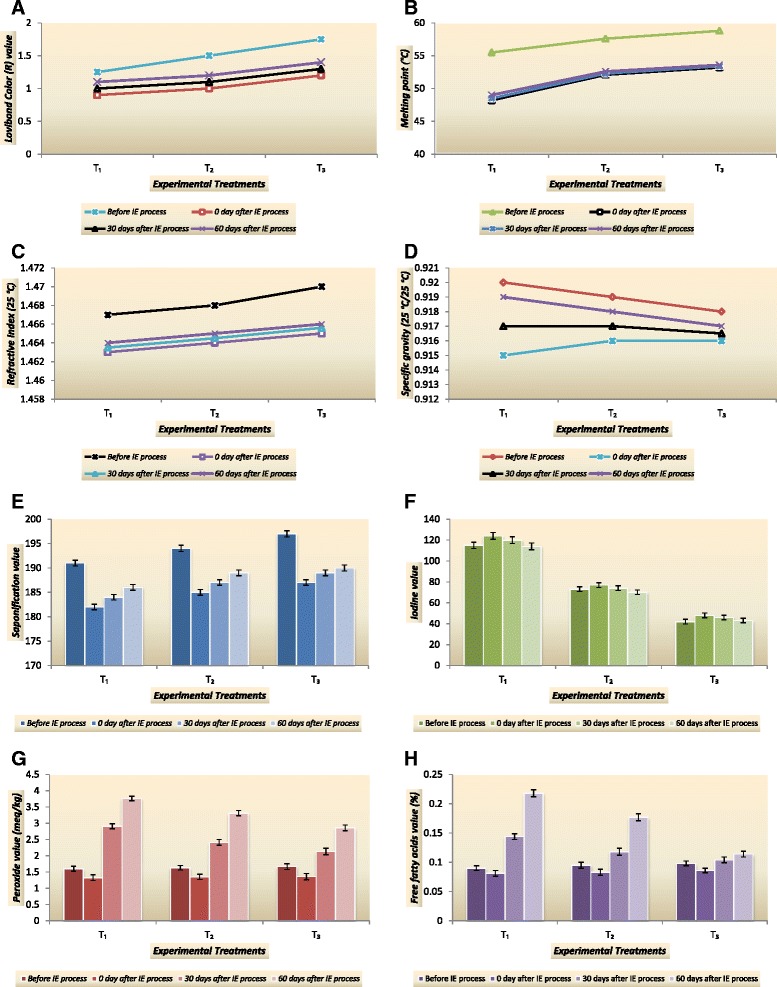
Effect of chemical interesterification process on physico-chemical characteristics and oxidative stability of CaO:FHCSO blends (**a** Lovibond color R value, **b** melting point, **c** refractive index, **d** specific gravity, **e** saponification value, **f** iodine value, **g** peroxide value and **h** free fatty acids value)

Lovibond color units, melting point, refractive index, specific gravity, saponification value and iodine value are usually used for the identification of physico-chemical characteristics the oils and their blends. Physico-chemical results of the study are reported in Fig. 1.

### Lovibond color R value

A steady decrease in Lovibond color R units was observed in all treatments after interesterification (Fig. 1a). The maximum decrease in color units was recorded for T_3_ (1.2 ± 0.4) as compared to its initial value (1.75 ± 0.6). The intensity of the color was seen to be lighter in T_1_, probably because of its refined state and was seen to have lowest color value of 1.1 ± 0.3 units. It was observed that color value of CaO:FHCSO blends increased non-significantly throughout the storage period *(p ≥ 0.05)*. Slight changes and darkening in color may be attributed to several factors such as S_3_ composition of blend, tocopherol contents, storage conditions and oxidative effects during storage [32, 33]. Chemical interesterification significantly decreased the tocopherol contents of vegetable oil samples [27]. Tocopherol loss is the most important and probably the only known disadvantage of chemical interesterification as α-Tocopherol has the highest vitamin E activity in vegetable oil blends. However, reducing the tocopherol content during interesterification does not inversely affect the oxidative stability of interesterified blends and tocopherol supplementation of interesterified oils with equal amounts of eliminated tocopherols can be successfully applied by related food industries [34].

### Melting point

Melting point of a fat has direct relationship with its degree of hardness and it can be used as criterion of purity. Fig. 1b shows the melting profile of CaO:FHCSO blends before and after interesterification. The melting profile of blends was directly proportional to the S_3_ contents coming from the FHCSO before interesterification. However, after interesterification, a sudden decrease in melting profile for all blends was noted. Maximum decrease in melting profile (7.3 °C) was for T_1_ which may be associated with extensive rearrangement of fatty acids among TAG and proportionally decrease of S_3_ contents in the CaO:FHCSO blend. A blend of 70 % hydrogenated canola oil, 10 % palm stearin, and 20 % canola oil had an initial dropping point of 37 °C, which dropped to 35 °C following 5 min of interesterification reaction and to 32 °C after 20 min, remaining constant thereafter [35]. Scientific studies confirmed that hardfat contents of a given sample are directly related to high-melting-point components of processing blends [23, 25, 36]. After interesterification, an absolute decrease in the melting point, ranging from 7–31 °C was detected for the different vegetable oil blends which can be explained by the decrease of the higher-melting S_3_ proportion [26]. The melting thermogram also confirmed the presence of interesterified product with lower melting point may be due the disappearance of the high melting TAGs [19, 24]. Furthermore, interesterification of fat blends with large amounts of hard stock (75 %) produced little change in the melting point [21, 22, 26] which also confirmed the results reported in the present study. During preservation period, a slight increase in melting profile of all treatments was recorded which may be linked to partial conversion of U_3_ to U_2_S, S_2_U and S_3_ through oxidative rancidity. It seems true that TAG type is the main determining factor for the attainment of blends with differentiated melting properties [36]. The research studies conclude that chemical interesterification of edible oil blends reduced melting points which are desirable physico-chemical properties for possible use as margarine, shortenings and confectionary fats [37, 38].

### Refractive index and specific gravity

The refractive index is a measure of extent of bending of light through a substance. The refractive index decreased slightly in all treatments with increasing conversion of S_3_ to S_2_U and U_2_S components after interesterification. Refractive index was affected by the chain length and number of double bonds molecules present in CaO:FHCSO blend. However, data on refractive index during storage reflected the stability of the oil blends up to 2 months and it ranged from 1.463 to 1.67 units (Fig. 1c). Increase in free acid contents, peroxide values and high storage temperature have been documented as responsible factors for slight increase in refractive index units of vegetable oil blends during storage [39, 40]. The changes in specific gravity of CaO:FHCSO blends before and after interesterification were monitored regularly and are presented in Fig. 1d. Slight decrease in specific gravity was recorded for all treatments after interesterification which is likely due to more double bonds nature of Cao:FHCSO blends. During storage, these values were found with slight increasing trend which may be attributed to the formation of S_3_ polymeric fractions.

### Saponification and iodine value

Slight decrease in saponification values could be noticed after the completion of interesterification process indicating the development of proportionally more U_2_S fractions in all treatments (Fig. 1e). T_1_ possessed lowest saponification value (182 ± 0.56) followed by T_2_ (185 ± 0.57) and T_3_ (187 ± 0.58) which can be relate to presence of highest unsaturated contents in T_1_ (93.5 ± 0.7), T_2_ (72.7 ± 0.6) and T_3_ (56.9 ± 0.5), respectively (Table 1). Saponification value is well-known index of the mean molecular weight of fatty acids comprising the triglycerides. The results indicate that triglyceride comprising of low molecular weight (short chain) fatty acids were more in T_3_ as compared to T_2_ and T_3_; therefore T_3_ showed high level of saponification value. The results further substantiated that saponification values were increased during storage for all treatments. For each CaO:FHCSO blend, increase in saponification value (1–2 %) was found after 60 days during storage period. Iodine value is considered as an index of the unsaturation, which is one of the most important analytical characteristic of oil. Data on changes in the iodine values of CaO:FHCSO blends are presented in Fig. 1f. The transition in iodine values was function of experimental CaO:FHCSO blends with concomitant decrease in unsaturated contents during blend formation while no change in degree of unsaturation was found after interesterification. It was also observed that iodine values decreased gradually during storage in the oil blends studied which may be due to decrease in double bonds by oxidative rancidity. Slow decrease in iodine value of oil blends may be due to induction period where fat was oxidized slowly showing initiation stage of auto oxidation reaction [33]. Rapid changes in iodine values of oil blends may be attributed to propagation of auto oxidation process where hydro peroxides are formed from free radicals in fatty acids generated in initiation stage or auto oxidation reaction. During the end of storage period slight change in iodine value was observed which might be due to termination stage of reaction [41].

### Oxidative stability of CaO:FHCSO blends

Lipids are composed of unsaturated and saturated fatty acids. The unsaturated parts are susceptible to oxidation when exposed to processing and storage and ultimately develop peroxide, hydro-peroxides, aldehydes, ketones, short chain fatty acids and finally bad smell. Oxidative changes in CaO:FHCSO blends were measured by peroxide and free fatty acid values which are displayed in Fig. 1.

### Peroxide value

Peroxide value can be used to determine the degree of deterioration and amount of oxidative rancidity of original oils blends. The changes in the peroxide values of selected CaO:FHCSO blends before interesterification, after interesterification and during storage can be seen in Fig. 1g. Peroxide values of T_1_, T_2_ and T_3_ were not significantly different from each other before interesterification *(p ≥ 0.05)*. The interesterified oils showed lower peroxide values than their non-interesterified counterparts in all CaO:FHCSO blends. Peroxide values of oil samples considerably increased up to 20 min of chemical interesterification, followed by a reduction at 30 min [27]. The reduction in peroxide values of vegetable oils after interesterification was also recorded by Basturk et al. [34] and Farmani et al. [42]. In the meanwhile, changes in peroxide values decreased as the concentration of FHCSO increased in the CaO:FHCSO blend during storage. T_2_ (3.31 ± 0.08 meq/kg) and T_3_ (2.86 ± 0.09 meq/kg) were seen well in oxidative stability than T_1_ (3.76 ± 0.07 meq/kg). However, the peroxide values of all CaO:FHCSO blends were recorded to be within the normal limits (5 meq/kg).

### Free fatty acids value

Free fatty acids occur in fats as a result of enzymatic hydrolysis by lipases, metal ions acting as free radicals or at an elevation of temperature. The free fatty acid values expressed in percent oleic acid of experimental treatments is depicted in Fig. 1h. Free fatty acids readily undergo oxidation, so their raised amount causes color and flavor deterioration of product. Free fatty acids of all the CaO:FHCSO blends were decreased after interesterification reaction. The decline in free fatty acids was may be due to the alkaline nature of sodium methylate used as catalyst. Sodium methylate is regarded as strong alkali and almost 70 % of the catalyst is used in neutralization of free fatty acids, only 30 % initiates and maintain the rearrangement reaction [43]. Free fatty acids in palm oil and palm olein blends were found quite low after chemical interesterification may be due to the reaction of alkaline sodium methylate catalyst with free acids [44]. The formation of free fatty acids in CaO:FHCSO blends were found to increase with increase in time of storage. T_1_ showed higher increasing trend for free fatty acids production as compared to T_2_ and T_3_ which can be explained on the basis of unsaturated TAG contents. Many authors have shown, however, that chemical interesterification can negatively influence the oxidative stability of fats and oils during storage. Non-interesterified and interesterified oils (canola, linseed, soybean, and sunflower) stored at 55 °C demonstrated little difference to lipid oxidation, whereas samples were found more stable at 28 °C [45]. Oxidative storage stability is strongly affected by lipid type and the lipids used for production [30]. The presence of non-TAG fraction in the interesterification products also lowers their resistance to oxidation [46] which seems true in present study as all experimental blends possessed less total TAGs at the end of storage period whereas the starting mixture showed the highest TAGs fraction. The optimum combination of hydrogenation and random interesterification can improve the oxidative stability of raw oils to expand the application in foods [47]. Moreover, the oxidative stability of the interesterified fats, which is reduced during storage, can be significantly improved using antioxidants [24]. Great importance is attributed to the bioactive components such as vitamin E and carotenoids to enhance the oxidative stability in food and biological system. However, the changes in findings may arise from the use of synthetic molecules slightly different from natural ones [48]. The supplementation of antioxidants in functional diet can protect human body from adverse events and dysfunction of metabolic syndrome because of the beneficial effects of these phytochemicals [49].

### Sensory evaluation of CaO:FHCSO blends

**Fig. 2:**
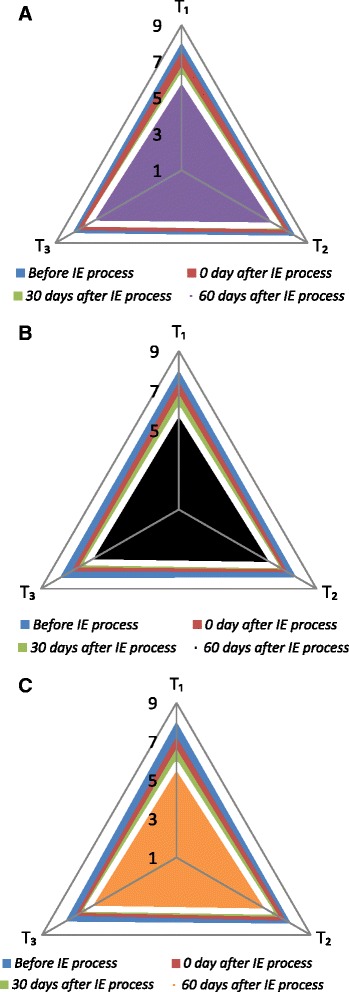
Effect of interesterification on sensory characteristics of CaO:FHCSO blends (**a** flavor attribute **b** appearance attribute **c** overall acceptability attribute)

Figure 2 represents the organoleptic ratings of CaO:FHCSO blends under different storage intervals. Original CaO:FHCSO blends before interesterification obtained most desired scores for flavor, appearance and overall acceptability attributes. On interesterification, CaO:FHCSO blends maintained their sensory acceptability and very slight variations in sensory scores were noted as compared to initial values. The results of sensory analysis showed the acceptability of a zero-trans fat shortening prepared by chemical interesterification of vegetable oil blends [50]. Refined olive oil and palm oil blends of varying proportions subjected to interesterification produced plastic fats similar in sensory properties to soft and package type margarine [51]. All sensory attributes were observed to decrease significantly as storage length increased from day 30 to 60-days. However, T_2_ and T_3_ obtained more sensory acceptability as compared to T_1_ during the whole storage period. Similarly, intensity of flavor was found to decrease following chemically interesterification of 100 % butterfat and 80 %–20 % butterfat–canola oil blends [52]. The trend of T_2_ and T_3_ desirability by sensory panel could be attributed to the composition and nature of fatty acids present in these CaO:FHCSO blends. The least desired sensory scores for T_1_ may be due to the higher amount of unsaturated contents present in CaO:FHCSO blend. In addition, oxidation of CaO:FHCSO blends during storage were observed to correlate negatively with the acceptability of functional fats. The lowest sensory values for T_1_ could also be linked to presence of aldehyde and ketone compounds which impacted the fishy like flavor in CaO:FHCSO blend at the end of study trial (after 60-days). Randomization was found to have no detrimental effect on the sensory acceptability [24]. It is also well known that the presence of U_2_S polymeric fractions with melting point in the range of 25 °C to 45 °C in developed functional fats is responsible for sensory properties of products at room storage temperature [53]. Most important was the 50 % CaO:50 % FHCSO blend (T_2_) which possessed the desirable TAG profile, physico-chemical and sensory characteristics coming from T_1_ and T_3_.

## Conclusions

The results of present study conclude that the marked reduction in trisaturated and triunsaturated contents with concomitant increase in desirable monounsaturated and diunsaturated contents can be achieved successfully for all CaO:FHCSO blends on interesterification. Results from this work will aid in the formulation of more healthy fat and oil products and may address a critical industrial demand in terms of formulation options for spreads, margarines and shortenings. Efforts should be continue for the production of functional lipids using medium-chain acids and long-chain polyunsaturated fatty acids present in locally available sources through exploring interesterification process as forefront lipid modification technology. Additional studies should be undertaken to determine the maximal shelf life of products supplemented with interesterified CaO:FHCSO blends and treatment of nutritional disorders through their absorption, metabolism and distribution pattern into biological tissues.
